# HMFITS-IDM: an episode-level decision model for interacting constraints, informed by four Japanese earthquake responses

**DOI:** 10.3389/fpubh.2026.1994092

**Published:** 2026-10-29

**Authors:** Nozomu Mandai

**Affiliations:** Faculty of Nursing Science, Tsuruga Nursing University, Tsuruga, Fukui, Japan

**Keywords:** decision-making, disaster response, feasible action set, health-service management, operational constraints, public health emergency management, sociotechnical systems

## Abstract

**Introduction:**

Operational decisions during disasters require more than a list of scarce resources: decision-makers must also assess which actions remain feasible. This paper proposes the Human, Material, Financial, Information, Time, and Safety/Quality-Integrated Decision Model (HMFITS-IDM) to support this assessment.

**Methods:**

The framework was specified through a conceptual, abductive approach and applied to eight decision reconstructions from four Japanese earthquake responses; the reconstructions draw on 14 academic and official sources. The procedure separates the decision frame from resource and feasibility appraisal, examines dependencies among actionable restrictions, compares candidate constraint sets against a fixed decision problem, and links the result to action and reappraisal. Each judgment is tied to a source location, and reported facts are distinguished from analytical judgments and proposed responses.

**Results:**

The Kumamoto example traces the reasoning from exhausted local receiving capacity, incomplete destination information, prolonged outdoor waiting, and patient-care requirements to a confirmed receiving arrangement and differentiated transport. Material is retained in the candidate explanation, but because smaller representations remain viable, minimality cannot be established. In other reconstructions, resource availability is distinguished from missing evidence, and one narrow transport-disruption decision can be explained by a single restriction. The Noto evidence also documents how financial uncertainty affected requests for support. Overall, the reconstructions support decision-specific explanations rather than a dominant bundle recurring within each earthquake pair.

**Discussion:**

HMFITS-IDM offers a procedure for making the basis of operational choices explicit and testing whether a smaller constraint explanation is sufficient. It accommodates competing and unresolved interpretations without requiring a bundle for every decision. In practice, the worksheet records why an option is restricted, what change could restore it, and when the decision should be reappraised. Reliability across independent coders, comparative utility, and prospective effectiveness remain to be evaluated.

## 1 Introduction

Operational decisions during disasters require more than identifying scarce resources. Decision-makers must determine which actions remain feasible, how interacting constraints restrict those actions, and which conditions can be changed within the available time. Disrupted infrastructure, incomplete information, and changing care needs make these judgments difficult. This study therefore uses disaster response as the setting for theory building.

Japanese earthquake responses are particularly informative because each event presented a different mix of operational constraints. The response to the 1995 Great Hanshin-Awaji Earthquake was marked by overloaded hospitals, uncontrolled patient flow, and failures in medical information and telecommunications (1–4). The 2011 Great East Japan Earthquake combined prolonged lifeline disruption, fuel shortages, communication limitations, radiation-screening requirements, and changing Disaster Medical Assistance Team (DMAT) roles (3, 5–7). The 2016 Kumamoto Earthquake required hospital evacuation and large-scale interfacility transfers supported by the Emergency Medical Information System (EMIS) and DMAT coordination (8–10). The 2024 Noto Peninsula Earthquake involved severe access constraints, damage to medical and welfare facilities, secondary evacuation, and support for a population with a high proportion of older adults (11–14).

Several established frameworks provide complementary perspectives on health-system organization, disaster response, resource allocation, and decision-making under constraints. Donabedian separates structure, process, and outcome (15), while surge-capacity frameworks and the World Health Organization (WHO) building blocks organize resources and health-system functions (16, 17). Resilience theory focuses on preparedness, continuity, adaptation, and recovery (18). The Systems Engineering Initiative for Patient Safety (SEIPS) 2.0 characterizes dynamic sociotechnical configurations (19). The Theory of Constraints seeks to identify the constraints that limit system performance and to focus improvement on them (20). Command-Control-Communications-Coordination, Staff-Stuff-Space-Special, and Tracking-Triage-Treatment-Transportation (CO-S-TR) supports rapid assessment during facility incidents (21). The Scarce Health Resource Allocation in Humanitarian Response Settings (SHARE-HRS) 4S^2^ model extends surge-capacity analysis, while the SHARE-HRS Decision Making Model addresses values, priorities, and decision processes in humanitarian resource allocation (22, 23). Bounded rationality and naturalistic decision-making further explain how decisions are made under uncertainty and time pressure (24, 25).

Each of these frameworks addresses part of the feasibility problem outlined above. The model proposed here brings together a decision frame, six-domain feasibility appraisal, comparison of actionable constraint sets, and a link to action and reappraisal. Beyond listing resource states, it aims to link each restricted option explicitly to the evidence or operational change needed to support action and to retain unresolved judgments for reappraisal. The comparison is limited to the frameworks discussed here and is not a systematic inventory of decision models.

This study proposes the Human, Material, Financial, Information, Time, and Safety/Quality-Integrated Decision Model (HMFITS-IDM). The HMFITS component appraises resources and feasibility conditions; the IDM component uses this appraisal to select or sequence feasible actions and to identify ways of restoring restricted options. This paper specifies the procedure, applies it to eight decision reconstructions from four Japanese earthquake responses, and develops propositions for future evaluation.

## 2 Conceptual approach and case comparison

### 2.1 Theory-building design

This conceptual study uses an abductive approach in which proposed distinctions are examined against case evidence and refined when they do not adequately represent reported decisions (26). Resource and health-system perspectives inform the capacity domains, whereas systems, constraints, and decision-making perspectives underpin the relationships among conditions and choices (15–25). The six-domain organization and the bundle rules are proposed analytical constructs; they have not been empirically validated.

The design separates four types of usable capacity—Human, Material, Financial, and Information—from Time and Safety/Quality, which determine whether an action can be implemented within the decision horizon while meeting the applicable requirements. Objectives, authority, and standards belong to the decision frame, whereas achieved results are treated as downstream outcomes. Comparing smaller constraint sets is intended to identify unnecessary or overlapping explanations without requiring a bundle for every episode.

Case evidence informs these distinctions. The Kumamoto account distinguishes exhausted local capacity from incomplete knowledge of capacity elsewhere (8), which supports separate appraisal of Material and Information. The Noto review describes how uncertainty about expense eligibility affected requests for welfare support (13), which indicates a role for Financial appraisal. Supplementary Table S6 connects the design choices to the source observations. It explains the present specification but does not record how the model was developed over time.

### 2.2 Case selection

The four earthquake responses were purposively selected to provide contrasts in geography and access, population vulnerability, compound hazards, and response-system maturity: Hanshin-Awaji (1995), Great East Japan (2011), Kumamoto (2016), and Noto Peninsula (2024) (1–14, 26). They do not form a representative sample and cannot be used to estimate how often particular constraint patterns occur.

The eight reconstructions cover two analytical topics for each earthquake (Supplementary Tables S3.1–S3.8). HA, GE, KU, and NO denote the four responses in chronological order. KU1 and KU2 are overlapping stages (receiving-site selection and transport allocation) of a single hospital evacuation; NO1 follows a named team’s transport task in Wajima. The other records describe regional, mission-level, or program-level problems; GE1, for example, concerns a specific Fukushima screening and evacuation mission rather than all continuity or public-health activities in 2011. The two records for each earthquake are not independent replications.

### 2.3 Source corpus and traceability

The corpus comprises 14 sources: 11 academic publications and three official reports. Sources were purposively selected from the disaster-medicine literature and from government and public-health publications, and the search was supplemented by citation tracing. Eligible content addressed hospital support, evacuation, transfer, continuity, information, logistics, or vulnerable-population support. The corpus includes research studies, field accounts, perspective articles, and system-development reports; it was not assembled through a systematic review.

The passages supporting the analysis were checked in the full texts. Supplementary Table S5 identifies the sources and their roles, and Supplementary Table S7 provides 37 entries with source locations, paraphrases, and limits of inference. Evidence IDs were assigned for this study; for example, R08a denotes reported evidence (R) from manuscript reference 8 (08), entry a in Supplementary Table S7. Passages in Japanese are paraphrased in English, and locators give the section names and the page numbers of the version consulted; where this differs from the published pagination (references 6 and 8), Supplementary Table S5 identifies the version used.

Records with different populations or timeframes were not combined into a single decision history. For example, the functionality survey in reference 9 and the regional transport report in reference 10 provide context for the transfer described in reference 8 but are not additional patient-level records of it. Later system accounts were not used to infer the resources available in 1995. Recommendations were distinguished from implemented actions, and relevant source discrepancies are recorded in Supplementary Table S8.

### 2.4 Analytical procedure and bundle rules

An episode is defined by a decision problem, the responsible actor (when identifiable), an objective, candidate actions, and a decision horizon or reappraisal point. Boundaries reported in the sources are distinguished from those set analytically. Here, “operational” refers to selecting or implementing actions within an episode; it implies neither unplanned action nor short-lived effects.

Supplementary Table S1 sets out the inputs, tests, and purpose of each stage. Bounding the episode prevents generalization to the whole disaster, and the frame separates authority and priorities from capacity. Recording the evidence provides the basis for classification, and feasibility screening identifies the restriction to be explained. Dependency and subset tests then examine whether a joint explanation is needed. Lastly, the action record links that explanation to a practical response.

Source-reported conditions and actions are distinguished from analytical interpretations, proposed responses, and unreported information. A component is exposed when evidence links it to an excluded, delayed, degraded, or unacceptable option. A component is buffered only when positive evidence shows that it does not restrict the option at the relevant site and time. A component that is neither exposed nor buffered is recorded as unresolved. Conditions not represented by the six domains are recorded as residuals. Components are further classified by role as fixed, enabling, or actionable. For example, a damaged building may be fixed, whereas receiving beds, transport, or supplies remain modifiable. Formal authority is recorded in the decision frame; deployable coordination capacity belongs to the Human domain.

Each candidate action is screened for resource feasibility, time feasibility, and its current ability to meet the Safety/Quality requirement set in the frame. Options are then classified as supported, constrained, or unassessed. Actions taken later are recorded separately from the information available before commitment. The Safety/Quality requirement remains fixed when constraint explanations are compared; omitting the Safety/Quality label from a candidate set does not remove the requirement.

A dependency statement identifies the affected option, the domain components, the supporting passages, and the mechanism by which one component changes another’s effect on the option. Co-occurrence alone does not demonstrate a dependency. Every retained domain must belong to a single connected set of dependencies; independent restrictions are considered separately. A component is horizon-actionable when an identified relaxation, substitution, or bypass can plausibly operate within the horizon and its implementation requirements are specified.

Candidate sets are compared against the same option restriction. All relevant proper subsets are listed or covered by an explicit screening rule. For each subset, the analysis states what the subset explains and what it leaves unexplained, while holding the source facts, background conditions, and decision frame constant. A set is inclusion-minimal when it satisfies the exposure, actionability, and connectedness tests and no proper subset adequately explains the same restriction. Inclusion-minimality does not mean choosing whichever explanation has the fewest domains. If a smaller representation or a necessary judgment remains unresolved, minimality is not established. These are qualitative explanatory tests; they do not estimate causal or statistical interactions.

The results are reported in five categories: supported candidates, conditional analytical minima, competing explanations, insufficient evidence, and decisions for which no multi-domain bundle is supported. A uniquely supported inclusion-minimal actionable set is termed dominant; the label is not assigned under uncertainty. Supplementary Table S2C records the KU1 comparisons, and Supplementary Tables S3.1–S3.8 report the results for all eight reconstructions.

Finally, each targeted restriction is linked to a reported action or proposed intervention, its expected effect on the option set, its implementation requirements, and a reappraisal trigger. Immediate outputs and downstream outcomes are recorded separately, so that what responders did is not confused with the explanation offered by HMFITS-IDM.

### 2.5 Testable propositions

Table 1 presents five testable propositions for future research.

**Table 1:** Testable propositions for future research.

Proposition	Observable implication	Candidate indicators/data sources	What would weaken it
P1. Feasible-action restriction	Interacting domains explain excluded, delayed, or re-sequenced actions better than single-domain accounts.	Episode records, timelines, EMIS or coordination logs, after-action reviews; comparison with single-domain explanations.	Most episodes are adequately explained by one independent shortage, or bundle coding adds no explanatory value.
P2. Minimal actionable interaction	A claimed minimum contains actionable, connected restrictions; no proper subset explains the same option restriction. Unresolved alternatives preclude a claim of dominance.	Counterfactual actionability tests; candidate-subset search; domain-removal challenges against a fixed explanatory target; blinded coding of competing, no-bundle, and insufficient-evidence cases.	Retained domains are fixed, independent, disconnected, or removable, or plausible alternatives are systematically omitted.
P3. Context-sensitive state transition	Domain states change when context, time, buffers, policy, or prior action changes.	Longitudinal logs, repeated scenarios, and before-after buffer or policy comparisons.	States do not vary with interpretable changes or vary inconsistently.
P4. Bundle-directed action	Actions directed at the retained bundle restore or preserve feasible options more efficiently than untargeted additions.	Simulation or prospective studies; time-to-option-restoration, resource use, and feasible-set change.	Untargeted actions perform as well as or better than bundle-directed actions, or bundle-directed action does not alter the feasible set.
P5. Health-service transferability	The same six domains and procedure represent new bounded disaster and health-service decisions without substantial residual conditions.	Independent coding in hospital, welfare-facility, and public-health scenarios; residual-domain rates and inter-rater agreement.	New settings consistently require additional domains or collapse decision-frame values and outcomes into HMFITS.

## 3 HMFITS-IDM specification and case illustrations

### 3.1 Conceptual architecture of HMFITS-IDM

Figure 1 summarizes the architecture. The operational context describes the setting, and the decision frame records objectives, authority, priorities, rules, and candidate actions. The HMFITS component appraises capacity and feasibility conditions. The IDM component then selects or sequences supported options and examines actionable restrictions when important options are constrained. Responses may involve reinforcement, substitution, reallocation, decoupling, or resequencing. If bundle membership is unresolved, a supported action or an intervention on a single restriction may still be appropriate, followed by reappraisal.

**Figure 1:**
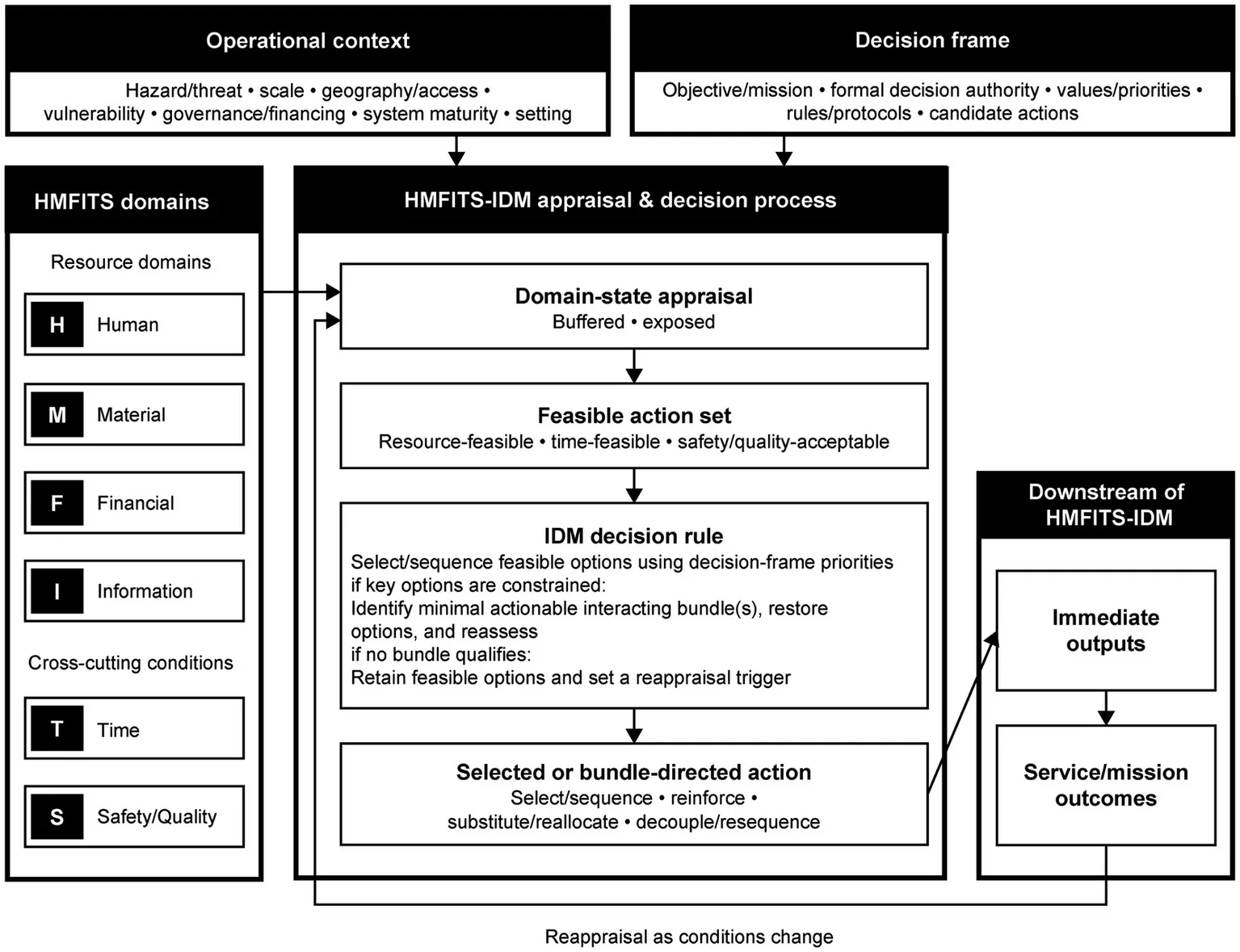
HMFITS-IDM decision architecture. The operational context and the decision frame inform six-domain appraisal, feasibility screening, candidate comparison, action, and reappraisal. Unknown states remain unresolved; a single restriction need not form a bundle. If minimum membership remains unresolved, a supported action or an intervention on a single restriction may proceed, followed by reappraisal. Outputs and outcomes are recorded separately. Preparedness shapes the context, frame, and available capability rather than constituting an additional domain. Section 2.4 and Supplementary Tables S1–S4 specify the tests and the treatment of unresolved cases.

Preparedness shapes response capability through pre-event training, deployment arrangements, and information-system preparation (3, 4, 7). These elements enter HMFITS-IDM through the operational context, the decision frame, and the resulting domain states rather than as a seventh domain. Trained staff and deployable reserves may buffer the Human and Material domains; practiced information sharing may buffer Information and reduce delays; and agreed authority and protocols inform the frame. Plans alone do not establish buffering: the planned capability must still be usable after the event. HMFITS-IDM therefore complements preparedness planning by appraising the conditions that apply to a particular decision.

The responsible decision-maker or a supporting analyst would complete the proposed worksheet (Supplementary Table S4). Its inputs are the objective, authority, horizon, candidate actions, applicable requirements, and current resource information. Its outputs are option-level feasibility judgments, a constraint explanation or an unresolved judgment, an action or sequence, and a reappraisal trigger. Supplementary Tables S2–S2D illustrate these fields with a retrospective example. The worksheet’s usability and operational benefit remain to be tested.

### 3.2 Working definitions of the HMFITS domains

Table 2 defines the domains. Appraisal is specific to the component, site, and time: a reserve at one receiving hospital does not establish buffering elsewhere. Communication hardware belongs to Material, whereas the availability and reliability of the information it carries belong to Information. Time concerns decision windows, implementation delays, sequencing, and sustainable duration; a timestamp for an outcome does not by itself indicate an exposed Time constraint. Safety/Quality concerns whether an action can currently meet the requirements set in the frame, not whether outcomes were achieved. The Financial domain is relevant when authorization, payment, procurement, or sustainment restricts an option; if financial conditions are not reported, their state remains unresolved.

**Table 2:** Working definitions of the HMFITS domains.

Domain	Working definition
H - Human	Available and deployable people, skills, leadership, role clarity, and coordination capacity. Formal authority is recorded in the decision frame.
M - Material	Physical assets and functional infrastructure, including facilities, beds, equipment, utilities, transport, communication hardware, and supplies. Fixed origin damage is distinct from modifiable receiving or supply capacity.
F - Financial	Capacity to authorize, mobilize, pay for, procure, and sustain the resources or arrangements needed for action.
I - Information	Timely, reliable, and shared information about needs, options, status, and consequences. Information about a resource is distinct from the resource itself and from the hardware used to communicate it.
T - Time	Decision windows, access and implementation delays, sequencing dependencies, and sustainable duration. Chronology alone does not establish an actionable restriction.
S - Safety/Quality	Current ability of an action to meet the safety, continuity, quality, dignity, or protection requirements specified in the decision frame. The requirement remains fixed during candidate comparisons.

### 3.3 Positioning HMFITS-IDM relative to selected frameworks

Table 3 compares HMFITS-IDM with seven directly relevant frameworks. Donabedian’s model, resilience theory, bounded rationality, and naturalistic decision-making are treated as conceptual foundations rather than direct structural comparators.

**Table 3:** Positioning HMFITS-IDM relative to selected frameworks.

Framework	Core focus	Proposed role of HMFITS-IDM in a bounded episode
Classical surge capacity/staff-stuff-structure (16)	Organizes personnel, supplies or equipment, and structural capacity for sudden demand.	Screens executable options across six domains and links episode-level constraint appraisal to action.
WHO health-system building blocks (17)	Diagnoses system functions, including workforce, information, products, financing, governance, and service delivery.	Adds a decision frame, feasible-action set, and episode-level constraint comparison linked to action.
SEIPS 2.0 (19)	Represents dynamic sociotechnical configurations, work processes, adaptation, and outcomes.	Adds a bounded choice set, domain-removal test, and explicit option-restoration step.
Theory of Constraints (20)	Focuses improvement on constraints limiting a system goal.	Tests whether a minimal interacting cross-domain bundle restricts feasible options within a specified horizon.
CO-S-TR (21)	Supports rapid facility incident assessment across command, logistics, and patient-care functions.	Provides a cross-domain procedure linking assessment to option selection, restoration, and reappraisal.
SHARE-HRS 4S^2^ model (22)	Organizes humanitarian surge resources and cross-cutting dimensions.	Translates capacity appraisal into episode-level feasibility screening and action across six domains.
SHARE-HRS Decision Making Model (23)	Makes allocation ideals, priorities, devices, context, and decision burden explicit.	Complements normative allocation by locating values in the decision frame and operational feasibility in HMFITS.

### 3.4 Great Hanshin-Awaji earthquake (1995)

HA1 and HA2 address early patient transfer and redistribution, respectively. Tanaka et al. describe shortages of personnel and supplies, hospital damage, uncontrolled patient flow, and the transfer of existing inpatients to accommodate new admissions (1). Miyamoto et al. identify the need for information on receiving facilities and transport, alongside failures of power and telecommunications (2). Later accounts describe how the system developed but do not show that DMATs were available in 1995 (3, 4).

The records support candidate relationships among capacity, information, coordination, and transport timing. However, they do not specify every local horizon or available bypass, so minimality remains unresolved. Physical restrictions are retained in the candidate explanations because the analysis does not assume that all Material components are fixed. Supplementary Tables S3.1, S3.2 link these judgments to the source passages and alternative explanations.

### 3.5 Great East Japan earthquake (2011)

GE1 concerns the Fukushima screening and evacuation mission reported by Ushizawa et al. (6). The account describes the requirements for protective equipment, screening, transport, and patient care. Almost all communication methods were available during this mission, unlike earlier missions, in which a lack of advance information prolonged face-to-face handovers. The availability of devices does not mean that information was complete, but the communication failure of the earlier missions cannot be assumed to apply to GE1. Exposure and actionability are insufficiently documented to establish a minimal bundle.

GE2 addresses the choice between continuing work, arranging relief, and withdrawing when needs outlast the planned DMAT deployment. Anan et al. describe some teams withdrawing when their scheduled period of activity ended, even though needs continued (7). This supports a Human–Time continuity explanation. It remains unresolved whether Safety/Quality contributes a separate condition or is already represented by the continuity requirement and the workforce state. Confirmed relief or handover is proposed here as an analytical response; its effectiveness as an intervention is untested. Evidence on the broader response is kept separate from these mission- and program-level records (3, 5).

### 3.6 Kumamoto earthquake (2016)

KU1 examines how a receiving hospital was found for patients of Kumamoto Central Hospital who were already outside after the main shock of 16 April 2016. Nagata et al. report that local admitting capacity was exhausted, that patients had waited outdoors for more than ten hours, and that rain was forecast for the evening; securing a safe place was the immediate priority (8). The coordination team used a community network to contact Himeno Hospital, which agreed to accept up to 150 patients. This sequence illustrates the difference between actual capacity and the information needed to confirm access to it.

Supplementary Table S2A traces the options before and after the receiving commitment. Further local admission was constrained by saturation, and an unconfirmed outside destination was not yet an executable arrangement. Once acceptance was confirmed, clinical grouping allowed a subgroup to be assigned to Himeno, while critically ill patients were sent elsewhere. The source also identifies Himeno’s new building and nursing home as alternative accommodation, so the old building was not indispensable. The regional supply of water, medicines, equipment, and oxygen further illustrates why Material cannot be equated solely with fixed origin damage (10).

The candidate {Material, Information, Time, Safety/Quality} represents capacity, acceptance information, urgency, and current care suitability, respectively. Supplementary Table S2B states the proposed dependencies and their source basis. Supplementary Table S2C compares all non-empty subsets of these four domains and separately screens candidates that include the Human or Financial domain. Human resources were used, but the sources do not show a staff shortage at the point of receiving commitment. Smaller explanations remain viable; for example, {Material, Information, Time}, with care requirements retained in the frame, is a competing representation. The four-domain candidate is therefore not a demonstrated minimum.

KU2 covers the overlapping allocation and transport stage. The account reports that 20 critically ill patients were transferred elsewhere and that 111 were assigned to Himeno, 71 by bus and 40 by ambulance, accompanied by five medical staff (8). Supplementary Table S2D traces the sequence from confirmed capacity to reception, including clinical grouping and the arrangement of transport modes and escorts. The reported times of first arrival (about 17:00) and admission (by 22:00) are treated as outputs rather than as deadlines set in advance. Because clinical cutoffs and the feasibility of alternative plans are not reported, the actual assignment is not treated as the only safe or optimal one.

### 3.7 Noto Peninsula earthquake (2024)

NO1 follows the transport task of the Tohoku University Hospital team in Wajima. On Day 2 of the team’s deployment, snow closed the main road, and patient transportation was not completed as planned; on Day 3, the patient transportation team directed other DMATs to transport inpatients by Self-Defense Force aircraft or other means (11). For the narrow purpose of explaining why the planned transport was disrupted, the reported road closure is interpreted as a single Material/access restriction. Staff, information, time, and safety remained relevant to implementing the alternatives, but their involvement does not indicate a second interacting exposed constraint.

NO2 concerns welfare placement at the program level. The official review reports shortages of staff and receiving facilities even after needs had been identified. It also reports delays in matching and, separately, initial difficulties in sharing transport information because the responsible prefectural department had not been clearly designated (13). The ministry describes how care workers available for dispatch from other prefectures were matched to staffing needs at facilities and 1.5-stage shelters (14). These records support candidate restrictions and responses but lack the common resident-level horizon needed to establish a minimum. A separate report from a receiving hospital describes available beds and staff but missing information during incoming transfers (12), which illustrates the need for appraisal specific to site and time.

The wider Noto review also reports that some local governments hesitated to request support because it was unclear whether disaster-relief funds would cover the operating costs of Disaster Welfare Assistance Teams. It further notes that raising the payment rate for hotels and ryokan (traditional Japanese inns) used as secondary shelters encouraged the industry to make rooms available (13). These program-level observations show that Financial appraisal is relevant, but they do not establish financial exposure for every transfer or quantify its effect.

### 3.8 Cross-case synthesis

Table 4 summarizes the analytical results. The results also show why each component must be appraised separately: fixed origin damage can coexist with usable receiving capacity; known needs do not guarantee that staff or places are available; and functioning communication equipment does not ensure adequate information. Across the records, the results include candidate configurations, competing explanations, unresolved minimality, insufficient evidence, and one narrow decision adequately explained by a single restriction. They do not show that the same dominant bundle recurs within each earthquake pair.

**Table 4:** Decision reconstructions and analytical results.

Reconstruction/resolution	Reported restriction or contrast	Analytical result
HA1: early transfer; regional	Physical capacity, receiving information, and coordination (1, 2).	Resource-inclusive candidates; incomplete information on local horizons and actionability leaves minimum membership unresolved.
HA2: redistribution; regional	Existing inpatients moved to accommodate arrivals; workforce, supply, and information limits (1, 3).	Multiple resource-inclusive explanations remain possible.
GE1: Fukushima mission	Screening and evacuation with most communication methods available (6).	Insufficient evidence of interacting exposed, actionable constraints to establish a minimum.
GE2: withdrawal; program	Scheduled deployment ends while needs remain (7).	{H, T} and {H, T, S/Q} are competing continuity explanations; local conditions remain incomplete.
KU1: receiving commitment	Local saturation, unconfirmed outside acceptance, prolonged outdoor waiting, and care requirements (8).	{M, I, T, S/Q} is a candidate; smaller representations remain viable.
KU2: transport allocation	111 patients to Himeno; 71 by bus and 40 by ambulance with five accompanying staff (8).	The reported allocation is traced, but neither minimum membership nor optimality is established.
NO1: transport disruption; named team	Snow closes the main road and patient transportation is not completed as planned; other DMATs are then directed to use aircraft or other means of transport (11).	A single Material/access restriction explains the narrow target; no multi-domain bundle is supported.
NO2: welfare placement; program	Shortages of staff and receiving places after needs are identified; separate matching and cost issues (13, 14).	Candidate capacity-matching explanations; without a common individual-level horizon, no single minimum can be established.

The eight reconstructions are not independent or equally complete decision records. HA, Hanshin-Awaji; GE, Great East Japan; KU, Kumamoto; NO, Noto. H, Human; M, Material; F, Financial; I, Information; T, Time; S/Q, Safety/Quality (one domain). Source links, competing explanations, and unresolved judgments are given in Supplementary Tables S3.1–S3.8.

## 4 Discussion

HMFITS-IDM links an explicit decision frame and feasibility appraisal to dependency analysis, comparison of constraint sets, action, and reappraisal. Its contribution is procedural; it does not introduce new resource or constraint categories. It complements, rather than replaces, existing resource, systems, and decision approaches (15–25). Table 5 distinguishes conceptually plausible implications from claims requiring further testing and from foundations supported by the existing literature.

**Table 5:** Evidential status of the implications of HMFITS-IDM.

Category	Implication/contribution	Basis and limits
1. Conceptually plausible implications	Combining episode boundaries, feasibility screening, candidate comparison, action, and reappraisal may make operational reasoning explicit.	Conceptual contribution illustrated through eight source-based reconstructions. The records do not establish uniquely minimal bundles or intervention effects.
2. Implications yet to be tested	Reliability of domain and bundle judgments; completeness of the six domains; option restoration, resource use, and decision consistency; transferability; corrective/transformative classification.	Independent coding, comparisons and prospective follow-up are needed (Table 1; Supplementary Table S4). Unresolved candidate comparisons are retained rather than treated as confirmed minima.
3. Implications supported by existing literature	Pre-event team preparation and information coordination contribute to response capability (3, 4, 7). Surge-capacity and SEIPS 2.0 frameworks address resource organization and sociotechnical interactions (16, 19).	This support concerns the underlying concepts, not the effectiveness or superiority of HMFITS-IDM.

The categories distinguish the basis of a claim; they are not mutually exclusive levels of certainty.

Minimality testing guards against overextended explanations: it identifies when a smaller set, a single restriction, or an unresolved judgment is more defensible than a larger bundle. The Kumamoto reconstruction illustrates why this matters even when no minimum is established. More information alone could not have created additional local beds, and the absence of confirmed outside acceptance did not show that outside capacity was lacking (8; Supplementary Tables S2A–C). The comparison therefore retains Material while leaving the independent roles of Time and Safety/Quality unresolved. Confirmed acceptance, patient grouping, and differentiated transport provide a source-linked action pathway without requiring minimum membership to be settled (Supplementary Table S2D). The added value lies in making explicit which explanation is insufficient, which action is supported, and what remains uncertain; any comparative benefit over simpler approaches remains to be tested.

For prospective evaluation, a temporarily corrective action is defined as one that restores or sustains feasible options without an enduring change to standing arrangements. A potentially transformative action is one intended to change capacity, coordination, or protocols beyond the response period. For example, a short-term redeployment of staff would be temporarily corrective, whereas retaining a revised staffing or referral arrangement after the response would be potentially transformative. These functions may coexist; intended transformation is not itself evidence of a lasting effect.

The worksheet records the intended function, the expected duration, dependence on temporary support, and the enduring change sought (Supplementary Table S4). Follow-up should record time-to-option-restoration, persistence at a specified date, and retention of changed arrangements. These proposed descriptive criteria have not been validated as a scale or used to classify the eight reconstructions. Uncertain cases remain unclassified, and longer duration does not imply greater rationality or benefit.

Future evaluation should first examine agreement between independent coders, disagreements about boundaries and component roles, residual conditions, and how often competing, unresolved, or no-bundle results occur. Scenario-based and prospective studies could then compare option restoration, resource use, decision consistency, and completion time with those achieved by simpler approaches. Such studies should include both cases with financial restrictions and cases with positive evidence of buffering. Table 1 sets out the relevant propositions and potential tests.

### 4.1 Limitations

The corpus is small, purposive, and heterogeneous. Several reconstructions are regional or program-level, and the paired stages overlap. Public accounts omit rejected alternatives, contemporaneous inventories, some actors, deadlines, and clinical criteria. The actions documented as executed therefore do not reveal the complete set of choices available beforehand. The evidence-to-design map (Supplementary Table S6) sets out the evidence behind the current specification but is not a real-time record of the model’s initial development.

Dependencies and minimality remain analytical judgments. Recording source locations and making comparisons explicit allow readers to re-examine these judgments, but they establish neither a causal structure nor agreement between analysts. Safety/Quality may partly summarize the effects of resources; whether it captures anything beyond those effects has yet to be evaluated. The Financial observations from Noto establish a boundary condition but not the prevalence or effect of financial constraints.

This study did not include an independent second coder, prospective deployment, or a comparative effectiveness assessment. The model does not prescribe universal ethical priorities, clinical cutoffs, utilities, or formal optimization; authorized practitioners retain responsibility for such judgments. Applications beyond disaster response require further study.

## 5 Conclusion

HMFITS-IDM is a proposed episode-level procedure that links the decision frame, six-domain appraisal, constraint comparison, and action. The reconstructions from four earthquake responses show how evidence can support a practical decision pathway while minimum membership remains unresolved. The contribution is an explicit, testable method for organizing these judgments; its reliability across independent coders, comparative utility, and prospective effectiveness remain to be established.

## Data Availability

The original contributions presented in the study are included in the article/Supplementary material, further inquiries can be directed to the corresponding author.
