# Supplementary material for: HMFITS-IDM: an episode-level decision model for interacting constraints, informed by four Japanese earthquake responses
Source: Front Public Health. 2026 Oct 29;14:1994092. doi: 10.3389/fpubh.2026.1994092 (PMC13754818; doi:10.3389/fpubh.2026.1994092)
Supplement: Supplementary file 1 [file Table_1.docx]

Supplementary Material 1

Coding rules, decision records and evidence for HMFITS-IDM

Reference numbers correspond to the reference list of the main manuscript. Table S1 specifies the analytical rules; Tables S2–S2D give the Kumamoto worked example; Tables S3.1–S3.8 record all eight reconstructions; Table S4 provides the prospective worksheet; and Tables S5–S8 document the sources, design rationale, evidence and source discrepancies.

1 **Coding and decision rules**

**Table S1. Inputs, tests and purposes of the HMFITS-IDM procedure.**

| **Stage** | **Record / test** | **Purpose** |
| --- | --- | --- |
| Bound the decision | Specify the decision, the actor (when known), the location, the options and the horizon. Identify analytical boundaries and overlapping or program-level records. | Defines the unit of analysis. |
| State the frame | Record the objective, authority, priorities and applicable care or safety requirements. Unreported deadlines and thresholds remain unspecified. | Separates decision criteria from operational conditions. |
| Register evidence | Record the source, page, section and paraphrase, and assign an evidence ID. Distinguish reported facts (R), interpretations (J), proposed responses (P) and unknowns (U). | Makes each judgment traceable. |
| Appraise components | Exposed (E): a supported restriction on a named option. Buffered (B): positive evidence of non-restriction at that site and time. Otherwise, unresolved (U). Record residual conditions and whether each component is fixed, enabling or actionable. | Distinguishes exposure from resource use or non-reporting. |
| Screen actions | Assess resource feasibility, timing and current care acceptability. Classify options as supported, constrained or unassessed, distinguishing the situation before and after commitment. | Identifies the option restriction to be explained. |
| Test actionability | Specify a possible change, substitution, bypass or sequence adjustment, its implementer, required resources and horizon. Appraise fixed origin damage separately from receiving capacity or supplies. | Limits intervention claims to executable changes. |
| State dependencies | Name the option, domain components, evidence and mechanism by which one component changes another’s effect on the option. Retained domains must form a connected structure. | Distinguishes interaction from co-occurring independent restrictions. |
| Fix the target | Use the same option exclusions, delays or acceptability contrasts for every candidate. Hold background facts and the decision frame constant. | Ensures that subsets are compared on the same basis. |
| Compare candidates | List relevant proper subsets or specify a screening rule. Record what each explains, what remains unresolved and the result. | Tests inclusion minimality without treating the number of labels as evidence. |
| Record the result | Distinguish supported candidates, conditional minima, competing explanations, insufficient evidence and cases with no supported multi-domain bundle. Dominance requires uniquely supported minimality and actionability. | Prevents forced bundle assignment or unwarranted claims of uniqueness. |
| Connect to action | Link a restriction to a reported action or proposed alternative, and record the expected change in options, the implementation requirements and a reappraisal trigger. | Connects the analysis to a decision. |
| Separate consequences | Record immediate outputs and downstream outcomes independently of domain coding. Identify changes requiring reassessment. | Prevents outcomes from being used to confirm the proposed bundle. |

2 **Evidence and interpretation conventions**

R denotes a source-reported condition or action; J, an analytical interpretation; P, a proposed response or comparison; and U, unreported or indeterminate information. E and B denote exposed and buffered domain states; when applied to a domain state, U denotes an unresolved state (neither E nor B). The terms fixed, enabling and actionable describe component roles; they are not additional domains.

The evidence IDs assigned in this study identify entries in Table S7; for example, R08a denotes reported evidence from reference 8, entry a, and R08a–c denotes entries R08a, R08b and R08c from that publication. Table S5 identifies the publications and the locations used; full bibliographic details are in the manuscript reference list.

H, Human; M, Material; F, Financial; I, Information; T, Time; S/Q, Safety/Quality.

3 **Worked example: Kumamoto receiving commitment and transfer**

**Table S2. Receiving commitment (KU1): decision boundary, target and analytical result.**

| **Field** | **Source-linked record** |
| --- | --- |
| Decision and actor | KU1: Nagata et al. (8) describe how the coordination team at the Kumamoto Prefecture Emergency Operation Center sought a receiving facility for patients already outside Kumamoto Central Hospital and contacted the president of Himeno Hospital (Table S7, R08a–c). |
| Boundary | 16 April 2016, from the capacity problem around noon through receiving commitment and patient grouping. This is an analytical boundary; an exact decision deadline is not reported. KU2 covers the overlapping transport and reception stage. |
| Fixed target | Explain the move from an unresolved local/outdoor situation to a confirmed out-of-prefecture receiving arrangement, considering capacity saturation, acceptance information, urgency and patient-care requirements. |
| Decision frame | The reported objective was prompt safe placement of patients and staff. Complete written priorities, the full set of alternative destinations and clinical thresholds are not available. Evening rain was forecast, but no latest safe time for transfer was specified. |
| Source roles | Direct account: Nagata et al. (8), advance-publication pp. 2–3. References 9 and 10 provide separate context on facility function and on regional coordination and supply; they are not additional records of the transfer of 111 patients. |
| Analytical result | {M,I,T,S/Q} is a supported candidate configuration. Its minimum membership is unresolved: {M,I,T}, with care requirements retained in the frame, is a smaller competing representation. Human exposure at commitment and Financial exposure are unreported. |
| Action link | The record supports confirmed acceptance, patient grouping, differentiated transport and reception. These actions can be interpreted without establishing a uniquely minimal bundle. |

**Table S2A. Option appraisal before and after the receiving commitment, based on Nagata et al. (8).**

| **Option / contrast** | **Reported basis or analytical contrast** | **Feasibility interpretation** |
| --- | --- | --- |
| A. Continue outdoor waiting | R: more than ten hours outside; rain forecast for the evening (R08a). | An interim situation that required resolution; it is not evidence that indefinite outdoor care was acceptable. A numerical safety cutoff is not available. |
| B. Seek further local admission | R: local admitting capacity was overwhelmed before noon (R08b). | Constrained by actual capacity (M); additional information alone would not create local beds. |
| C. Use an unconfirmed outside destination | R: direct communication between prefectures had not established acceptance (R08b–c). | Not yet supported as an executable option. Unknown acceptance is distinct from a physical absence of outside capacity. |
| D. Confirm Himeno and allocate a suitable subgroup | R: contact through a community network secured acceptance of up to 150; 111 were assigned after triage, with critically ill patients sent elsewhere (R08c, R08e). | A receiving arrangement supported after confirmation and grouping. Suitability for every patient is not implied. |
| E. Assign the entire narrative cohort of 190 to Himeno | J: a capacity contrast, not an option that responders were reported to have considered (R08a, R08c, R08e). | The 150-person commitment does not cover 190. The comparison does not resolve the source’s separate 190/200 discrepancy (Table S8). |
| F. Rely only on the old Himeno building | J: a component-necessity comparison. The source names the new building and nursing home as alternatives (R08d). | Usable accommodation may be necessary without one particular building being indispensable. |

**Table S2A (continued). Component appraisal at the KU1 receiving-commitment stage.**

| **Domain** | **Evidence at receiving commitment** | **State / role and uncertainty** |
| --- | --- | --- |
| Human | Staff were used for coordination, triage, escort and reception (R08c, R08e, R08f). | Enabling capacity is reported; local exposure is U. The regional logistics delay (R10b) is not a matched staffing record. |
| Material | Local capacity was insufficient; outside space and transport were mobilized (R08b–f). | E for local placement; an outside-capacity bypass is documented. Origin damage and receiving resources are separate components. |
| Financial | No payment, procurement or reimbursement restriction for this commitment is reported in reference 8. | U, not B. Financial observations elsewhere do not establish KU1 exposure. |
| Information | Acceptance information was inadequate until network contact secured a commitment (R08b–c). | E for the unconfirmed destination; contact provided a bypass. The number of patients accepted does not establish care capability for every patient. |
| Time | Prolonged waiting, forecast rain and a reported journey time of under two hours made timing relevant (R08a, R08c). | Waiting and sequencing warrant appraisal; an exact deadline and an independently necessary Time contribution remain unresolved. |
| Safety/Quality | Safe placement, clinical grouping and differentiated transport modes were reported (R08a, R08e). | Current care suitability is relevant. Whether it is non-redundant, given the resource conditions and the fixed frame, is unresolved. |

**Table S2B. Dependency reasoning and evidentiary limits.**

| **Link** | **Option-specific reasoning** | **Evidence (Table S7)** | **Interpretation / limit** |
| --- | --- | --- | --- |
| M–I | Placement at an outside site requires both actual capacity and confirmed access. Information cannot create a local bed once capacity is exhausted. | R08b–c | J: linked capacity and acceptance. Contact was established that day; the capacities of the alternative destinations are unknown. |
| I–T | Unresolved acceptance can delay the arrangement of care while patients remain outside. | R08a–c | J: timely confirmation serves the same-day objective. The time saved by contact is not quantified. |
| M–T | Receiving location and transport affect how promptly placement can be implemented. | R08c, R08e | J: the reported journey and transport sequence support the link but do not establish an exact patient-level deadline. |
| M–S/Q | Space alone does not establish suitability for every patient; clinical groups used different facilities and modes. | R08d–e; separate context R09a | J: current care capability relates capacity to acceptability. A resource-plus-frame representation remains a competing representation. |
| T–S/Q | Continued outdoor waiting conflicted with the immediate safe-placement objective. | R08a | J: urgency and acceptability are linked; it is not established that each label is independently necessary. |
| H with I/M | Staff were responsible for contact, triage, escort and reception. | R08c, R08e, R08f; regional context R10b | Reported implementation, not proof of a separate exposed Human constraint at commitment. |

All 15 non-empty subsets of {M,I,T,S/Q} are represented below. The remaining 16 H-containing subsets without F and 32 F-containing subsets are grouped because exposure or actionability is unresolved. This enumeration does not represent 63 completed empirical tests. Source facts, care requirements and the decision frame remain fixed during each comparison.

**Table S2C. Candidate comparisons for the fixed KU1 target.**

| **Candidate / family** | **Comparison against the fixed target** | **Result** |
| --- | --- | --- |
| {M}; {I}; {T}; {S/Q} | {M} covers capacity, not acceptance information; {I} covers acceptance information, not capacity. {T} and {S/Q} cover urgency and acceptability, respectively, not the capacity-and-acceptance contrast. | No single-domain explanation is supported for the full KU1 target. Single-restriction actions may still be justified. |
| {M,I} | Represents capacity saturation and confirmation of outside access. | Explains the receiving-access contrast; whether T or S/Q is indispensable for explaining the broader target remains unresolved. |
| {M,T} | Represents capacity and delivery timing. | Does not explain the reported inability to ascertain acceptance (R08b–c). |
| {M,S/Q} | Represents usable care capacity and acceptability. | Does not explain the lack of confirmed acceptance. |
| {I,T} | Represents timely communication. | Does not account for exhausted local physical capacity (R08b). |
| {I,S/Q} | Represents confirmation and care suitability. | Does not account for the exhaustion of actual local capacity. |
| {T,S/Q} | Represents urgency and acceptability. | Omits capacity saturation and unconfirmed acceptance. |
| {M,I,T} | Covers capacity, confirmation and urgency with care requirements retained in the frame. | A competing representation; a separate non-redundant S/Q contribution is not established. |
| {M,I,S/Q} | Covers capacity, confirmation and acceptability with a same-day horizon. | A competing representation if no separate actionable Time restriction is established. |
| {M,T,S/Q} | Covers capacity, timing and care suitability. | Does not explain the initially unconfirmed acceptance. |
| {I,T,S/Q} | Covers information, timing and care suitability. | Insufficient for the full target because it leaves the exhaustion of local capacity unrepresented. |
| {M,I,T,S/Q} | Represents the combined restrictions through the links in Table S2B. | Supported candidate, not an established minimum; smaller competing representations remain. |
| H-containing sets without F | Sixteen subsets contain H and any combination of M, I, T and S/Q. | Exposure/actionability screen unresolved: observed staffing does not establish a local Human shortage. |
| F-containing sets | Thirty-two subsets contain F and any combination of the other five domains. | Exposure/actionability screen unresolved: no KU1-specific financial restriction is reported. Includes H+F sets. |

The evidence supports a resource-inclusive candidate for KU1, but some smaller representations cannot be ruled out. Minimum membership is therefore left unresolved.

**Table S2D. Reported actions in Nagata et al. (8), analytical links and proposed reappraisal.**

| **Stage** | **Reported action** | **Analytical link** | **Proposed check / reappraisal** |
| --- | --- | --- | --- |
| Receiving commitment | R: network contact and Himeno’s acceptance of up to 150 (R08c). | J: turns an unconfirmed outside destination into a confirmed arrangement. | P: verify acceptance, care capability and responsible contact; reconfirm after changes. |
| Patient grouping | R: clinician triage; 20 critically ill patients sent elsewhere; 111 to Himeno (R08e). | J: combines receiving capacity with patient requirements. | P: apply responsible clinicians’ criteria; additional beds alone do not establish suitability. |
| Mode and escort allocation | R: 71 by bus, 40 by ambulance, five accompanying staff; multiple agencies supplied transport (R08e). | J: specifies how the receiving arrangement is implemented. | P: verify vehicles, escorts and receiving readiness; alternative transport mixes remain unassessed. |
| Reception | R: staff mobilization and allocation of patients across buildings/facilities according to their status (R08d–f). | J: space and staff allow the care pathway to be implemented. | P: reassess if arrivals, needs, utilities or staffing change. |
| Outputs | R: first arrival about 17:00; admission by 22:00; no adverse transport events reported (R08f). | J: describes implementation after commitment, not a criterion proving bundle minimality. | P: record actual timing and adverse-event ascertainment separately from prior assumptions. |
| Continuity and return | R: staff from the originating hospital supported care; patients returned after repairs (R08f). | J: a later care stage with its own conditions. | P: reassess after a new shock, deterioration, capacity change or restoration of the original facility. |

4 **Eight decision reconstructions**

HA, GE, KU and NO denote the Hanshin-Awaji, Great East Japan, Kumamoto and Noto responses, and the numbers 1 and 2 distinguish the two reconstructions for each. The records use the same fields but differ in resolution. KU1 and KU2 overlap; several other records describe regional or program-level problems. Candidate sets are analytical explanations, not estimates of unique minimal bundles. Evidence IDs refer to entries in Table S7.

**Table S3.1. HA1 — Receiving destination and transport mode after the Hanshin-Awaji Earthquake.**

| **Field** | **HA1 decision record** |
| --- | --- |
| Boundary / sources | Regional early-transfer problem based on Tanaka et al. (1) and Miyamoto et al. (2), during the transport peak in the first four days (R01a–b; R02a–b). The period of 17–20 January is an analytical window, not a patient deadline; no single hospital decision is reconstructed. |
| Options / feasibility | R: ground transfers and limited helicopter use; backup hospitals received patients. Local care was constrained at affected facilities. The availability of a particular vehicle, bed or communication channel for each patient is U. |
| Domain appraisal | H: regional workforce/coordination restriction, local state U. M: facility, transport and communication restrictions. I: receiving/transport information deficits. T: early urgency, individual actionability U. S/Q: care requirements, individual thresholds U. F: U. |
| Dependencies | J: destination information guides movement only where transport and receiving capacity exist (I–M); personnel coordinate movement (H–I/M). Constituent restrictions are documented, but each local dependency is not. |
| Candidate comparison / result | {M,I}, {H,M,I} and time-inclusive explanations remain possible for different local decisions. An explanation that omits Material, such as {H,I,T}, is unsupported. Incomplete information on horizons and bypasses leaves minimality unresolved. |
| Action / reappraisal | R: transfers to backup hospitals. P: confirm receiving capacity and an available transport mode, support coordination and reassess routes and capacity. These proposals do not imply that such options were available at the time. |

**Table S3.2. HA2 — Redistribution and support for overloaded hospitals.**

| **Field** | **HA2 decision record** |
| --- | --- |
| Boundary / sources | Regional early load-balancing problem, overlapping HA1. Tanaka et al. (1) describe the transfer of existing inpatients to accommodate new arrivals (R01b); Homma (3) retrospectively describes uneven patient loads and the late arrival of teams (R03a). |
| Options / feasibility | R: local admission and transfer of existing patients. P: diversion, redistribution or external reinforcement requires local availability; not all of these are documented alternatives. |
| Domain appraisal | H: workforce shortage and late assistance. M: impaired facilities and supplies. I: deficient interhospital information. T: demand accumulation, local horizon U. S/Q: care requirements, current patient-level capability U. F: U. |
| Dependencies | J: redistribution combines knowledge of capacity with the ability to move and care for patients (I–M/H). Uneven loads corroborate the problem but do not isolate information as its sole cause. |
| Candidate comparison / result | {I} does not explain staff or physical shortages. {H,M,I} and horizon-specific extensions are candidates; neither Material sufficiency nor a single minimal actionable set is established. |
| Action / reappraisal | R: transfers created room for arrivals. P: compare local load with confirmed outside capability and transport while maintaining care. The later development of DMATs (R04a) provides context; DMATs were not an option in 1995. |

**Table S3.3. GE1 — Fukushima screening and evacuation mission.**

| **Field** | **GE1 decision record** |
| --- | --- |
| Boundary / sources | Ushizawa et al. (6): Tokyo Medical and Dental University Hospital (TMDUH) Mission 4 with other DMATs and radiation-response headquarters (R06b). The discrepancy in the source’s relative day count is retained in Table S8; no corrected date is assigned. |
| Options / feasibility | R: screening and evacuation of elderly and disabled patients using protective equipment, dose-meters and transport. Remaining in place and unscreened transfer are analytical contrasts rather than fully documented choices. |
| Domain appraisal | H/M: staff, equipment and transport used; shortages not established. I: communication methods largely operable, complete patient/destination information U. T: exact actionable restriction U. S/Q: screening and care requirements. F: U. |
| Dependencies | J: screening and transport require staff and equipment to meet protection and care requirements. Implementation dependency does not itself establish interacting exposed constraints. Handover delays in the earlier missions (R06c) are a separate matter. |
| Candidate comparison / result | {I,T,S/Q} is not confirmed because information exposure and actionable Time are not established for this mission. {H,M,S/Q} describes implementation, not proven independent restrictions. The evidence is insufficient to establish a minimal bundle. |
| Action / reappraisal | R: screening followed by evacuation. P: verify screening capacity, patient and destination information and transport conditions; reassess if conditions change. Broader food and water measures (R05b) fall outside this mission. |

**Table S3.4. GE2 — Continue, relieve or end DMAT work.**

| **Field** | **GE2 decision record** |
| --- | --- |
| Boundary / sources | Anan et al. (7) describe withdrawal after the planned 48–72 hours of activity (R07a). This is a program-level withdrawal/handover problem; individual teams and a complete timed handover record are not identified. |
| Options / feasibility | R: some teams withdrew when their scheduled activity ended. P: continued operation, confirmed relief, staged handover or transfer of care requires verification at each location. |
| Domain appraisal | H: team loss with ongoing needs. T: scheduled duration versus continuing demand. S/Q: continuity requirement. M: endurance resources may restrict action (R03b), local status U. I: ongoing-needs information required, exposure U. F: U. |
| Dependencies | J: ending deployment changes care availability if the workforce is not replaced (H–T). It remains unresolved whether S/Q adds a separate condition or is represented by the continuity frame. |
| Candidate comparison / result | {H,T} and {H,T,S/Q} are competing representations; {I,T,S/Q} omits team loss. Material-inclusive explanations remain possible where endurance resources are restrictive. Unique minimality is not established. |
| Action / reappraisal | P: establish current needs, team endurance and confirmed relief before relying on continued service. The source documents the problem but not the comparative effectiveness of this remedy. Reassess if relief, supplies or needs change. |

**Table S3.5. KU1 — Kumamoto Central receiving-site commitment.**

| **Field** | **KU1 decision record** |
| --- | --- |
| Boundary / sources | 16 April 2016, from around noon through receiving commitment and patient grouping; Kumamoto coordination team and Himeno leadership. Source: Nagata et al. (8), R08a–e. The detailed record is in Tables S2–S2D. |
| Options / feasibility | Outdoor waiting required resolution; local admission was capacity-constrained; outside acceptance was initially unconfirmed. A commitment to accept up to 150 patients preceded the assignment of 111 to Himeno. |
| Domain appraisal | M/I: distinct documented restrictions. T/S/Q: urgency and care suitability relevant, independent necessity unresolved. H: reported enabling resources, exposure U at commitment. F: U. |
| Dependencies | J: M–I, I–T, M–T, M–S/Q and T–S/Q connect the receiving arrangement to capacity, confirmation, urgency and care requirements (S2B; R08a–e). |
| Candidate comparison / result | Table S2C compares 15 subsets of {M,I,T,S/Q} and screens H/F-containing families. {M,I,T,S/Q} is a candidate; smaller frame-based representations remain viable. No minimum is uniquely established. |
| Action / reappraisal | R: Himeno acceptance, grouping and transport. P: continue checking acceptance, capacity and care, and reassess if conditions change. Later success is not used to determine the initial classification. |

**Table S3.6. KU2 — Patient-group, transport-mode and reception allocation.**

| **Field** | **KU2 decision record** |
| --- | --- |
| Boundary / sources | From receiving acceptance on 16 April through same-day reception. Clinicians from the originating hospital and receiving teams implemented the arrangements. Overlaps KU1. Source: Nagata et al. (8), R08c–f. |
| Options / feasibility | R: 20 critically ill patients elsewhere; 111 to Himeno, with 71 by bus, 40 by ambulance and five accompanying staff. Alternative all-bus or all-ambulance plans are unassessed. |
| Domain appraisal | H/M: escorts, vehicles, staff and space were used. I: triage and acceptance information. T: staged arrival, exact prior deadline U. S/Q: clinical and transport fit. F: U. Resource use alone does not establish exposure. |
| Dependencies | J: the appropriate destination and transport mode depend on patient requirements, as identified through triage, and on available transport/care capability. The record does not supply a complete alternative-capacity analysis. |
| Candidate comparison / result | {M,S/Q} represents patient–mode matching, but {M} plus the fixed clinical frame is a competing representation. H/I/T additions require evidence of exposure. Minimum membership remains unresolved. |
| Action / reappraisal | R: differentiated transport, reception and continuity support. P: reassess vehicles, escorts, destination fit and readiness as conditions change. The reported allocation is not established as uniquely optimal. |

**Table S3.7. NO1 — Wajima route substitution.**

| **Field** | **NO1 decision record** |
| --- | --- |
| Boundary / sources | Tohoku University Hospital DMAT, from Day 2 through subsequent transport arrangements at Wajima Municipal Hospital, during a deployment beginning on 6 January 2024. Source: Egawa et al. (11), R11a–b. |
| Options / feasibility | R: snow closed the main road and patient transportation was not completed as planned. On Day 3, the patient transportation team directed other DMATs to use Self-Defense Force aircraft or other means of transport. Thirty inpatients were transported by a Self-Defense Force aircraft on Day 4. Other routes, or waiting, would require local appraisal. |
| Domain appraisal | M: documented route closure, with substitution reported. H/I: coordination and support used, separate shortages not established. T: delay reported, independent actionable restriction U. F/S/Q: exposure U; care requirements remain in the frame. |
| Dependencies | J: for the narrow purpose of explaining why patient transportation was not completed as planned, the reported road closure is interpreted as a single Material/access restriction. The record does not establish a second interacting exposed domain necessary for that explanation. |
| Candidate comparison / result | For this target, a single Material restriction is sufficient; no multi-domain bundle is supported. This does not imply that all conditions of the wider evacuation system were adequate. |
| Action / reappraisal | R: transport substitution. P: reassess weather, routes, patient requirements and capacity. Beds or staff available at the separate hospital in R12a are not evidence of capacity at Wajima. |

**Table S3.8. NO2 — Welfare placement after needs were identified.**

| **Field** | **NO2 decision record** |
| --- | --- |
| Boundary / sources | Official working-group review (13) and MHLW (14): program-level movement from 1.5-stage shelters to receiving care facilities, including care-worker dispatch matching (R13a, R13c; R14a–b). Individual residents, a common deadline and complete options are not recorded. |
| Options / feasibility | R: some people remained in shelters despite identified needs because staff and places were insufficient. Receiving arrangements and, separately, care-worker dispatch matching were pursued. P: supported placement or reinforced interim care requires local verification. |
| Domain appraisal | H/M: shortages reported. I: some needs known, other matching/information handoffs deficient. T: prolonged stays, individual actionability U. S/Q: continuing-care requirements. F: uncertainty about the costs of requesting support concerns a separate subdecision in the wider program (R13b). |
| Dependencies | J: care placement requires a staffed place suited to needs. The aggregate report does not establish simultaneous H/M restriction for the same resident; information and expense issues may concern different decisions. |
| Candidate comparison / result | {H,M} is a candidate capacity-matching explanation. H-only and M-only restrictions remain possible at different sites. I/F-inclusive sets require matched evidence. Connected minimality remains unresolved. |
| Action / reappraisal | R: care workers available for dispatch were matched to staffing needs at facilities and 1.5-stage shelters. P: verify needs and preferences, a staffed place, transport and relevant authority/cost arrangements; reassess if conditions change. |

5 **Practical decision-episode worksheet**

Table S4 is a proposed reporting aid for an authorized decision-maker or supporting analyst. The worked example (Tables S2–S2D) illustrates how its core fields are completed. The worksheet is not a clinical triage protocol, and its usability and effectiveness have not been validated.

**Table S4. Prospective worksheet: appraisal, action and follow-up.**

| **Step** | **Record** | **Question / check** |
| --- | --- | --- |
| 1. Bound | Decision, actor/authority, location, time, horizon or trigger, source references or identifiers. | What is being decided now? Unreported values remain U; completion time is not a prior deadline. |
| 2. Frame | Objective, priorities, applicable requirements and candidate actions. | Which requirements apply to all options? Standards remain separate from current capability. |
| 3. Appraise | For each domain (H, M, F, I, T, S/Q): evidence, E/B/U, component role, residual conditions and uncertainty. | Which component restricts which option? Resource use, non-mention or reserves elsewhere do not establish local buffering. |
| 4. Screen | Resource and time feasibility, current care acceptability; supported, constrained or unassessed; before/after commitment. | Which options are supported at this point? Missing information does not prove that an option is impossible. |
| 5. Compare | Fixed target, dependencies and evidence, candidates and subset reasons, unresolved alternatives and result. | Does a smaller explanation or a single restriction suffice? Unresolved relevant alternatives prevent a claim of minimality. |
| 6. Act | Chosen action or sequence, targeted restriction, expected change in options, implementation resources/authority and responsible actor. Intended function: temporarily corrective, potentially transformative or mixed; expected duration; reliance on temporary support; enduring change sought; uncertainty. | Is the action executable and consistent with the frame? What is the action intended to achieve, as distinct from how long it is expected to last? |
| 7. Reappraise | Immediate output, downstream outcome, missing information, trigger and responsibility for reassessment. Time-to-option-restoration; follow-up date; persistence of effects; retained changes to arrangements. | What change could invalidate the choice or reveal another option? At follow-up, has the intended change persisted? |

The action-function fields are proposed for prospective assessment and were not applied retrospectively to these eight records. The intended function is recorded separately from observed persistence; insufficiently documented cases remain unclassified.

6 **Source index**

The 14 sources are identified below; reference numbers match the manuscript. Full bibliographic details are provided in the manuscript reference list. Differences between sources in setting, date and population are retained, and recommendations are distinguished from implemented actions.

**Table S5. Publications, source locations and analytical roles.**

| **Reference / publication** | **Locations used** | **Source type / analytical role** |
| --- | --- | --- |
| (1) Tanaka et al. (1998) Overview of evacuation and transport of patients following the 1995 Hanshin-Awaji earthquake. | pp. 439, 442–443 | Retrospective multi-hospital patient-record study R01a–b; HA1–HA2; descriptive evidence of transport and load restrictions. |
| (2) Miyamoto et al. (1999) Great earthquakes and medical information systems, with special reference to telecommunications. | pp. 254–257 | Hospital questionnaire / information-systems case report R02a–b; HA1; communication hardware, information needs and phase changes. |
| (3) Homma (2015) Development of the Japanese National Disaster Medical System and experiences during the Great East Japan Earthquake. | pp. 53, 55, 60 | Review and disaster-system/evacuation account R03a–b; system context; retrospective explanatory claims remain attributed to the source. |
| (4) Kondo et al. (2009) Establishing disaster medical assistance teams in Japan. | pp. 556–558, 560–562 | Special report on the establishment and operation of DMATs R04a; later system-development context, not evidence of a 1995 DMAT option. |
| (5) Saito and Kunimitsu (2011) Public health response to the combined Great East Japan Earthquake, tsunami and nuclear power plant accident: perspective from the Ministry of Health, Labour and Welfare of Japan. | pp. 7–8 | Ministry perspective R05a–b; broad continuity and public-health context, not a single-site episode. |
| (6) Ushizawa et al. (2013) Needs for disaster medicine: lessons from the field of the Great East Japan Earthquake. | pp. 3–4 (article version paginated 1–5) | Four-mission field account R06a–c; mission-specific tasks and communication. |
| (7) Anan et al. (2014) Experience from the Great East Japan Earthquake response as the basis for revising the Japanese Disaster Medical Assistance Team (DMAT) training program. | pp. 477–478 | Training-program revision based on reports/literature R07a–b; duration, withdrawal, evacuation and information-training lessons. |
| (8) Nagata et al. (2017) Successful hospital evacuation after the Kumamoto Earthquakes, Japan, 2016. | pp. 2–3 (advance-publication version paginated 1–5) | Specific hospital evacuation / receiving-hospital account R08a–f; direct KU1–KU2 account. |
| (9) Achour and Miyajima (2020) Post-earthquake hospital functionality evaluation: the case of Kumamoto Earthquake 2016. | pp. 1673–1674, 1687, 1690 | Questionnaire study of hospital functionality R09a; the original survey provides context on hospital functionality. Its citation of reference 8 is not independent corroboration. |
| (10) MHLW / DMAT Secretariat (2016) Kumamoto Earthquake Report [title translated]. | pp. 3–4, 7, 11, 17–20 | Official meeting report, Japanese, 9 September 2016 R10a–e; regional information, logistics, supply and exceptions to continuity. |
| (11) Egawa et al. (2024) The 2024 Noto Peninsula Earthquake and the strategy of medical assistance from the Tohoku University Hospital. | pp. 47–48 | News and Views / team-response account R11a–b; named Wajima task sequence for NO1. |
| (12) Uramoto et al. (2024) Initial response to the 2024 Noto earthquake by the university hospital closest to the disaster area. | pp. 3, 5 | Single receiving-hospital observational account R12a–b; distinct hospital-level buffering and information boundary checks. |
| (13) Central Disaster Management Council working group (2024) Report on disaster response based on the 2024 Noto Peninsula Earthquake [title translated]. | pp. 94, 101 | Official response review (November 2024), Japanese R13a–d; welfare placement, secondary evacuation and Financial boundary conditions. |
| (14) MHLW (2024) Special feature: the Ministry of Health, Labour and Welfare’s response to the 2024 Noto Peninsula Earthquake. Annual Health, Labour and Welfare Report 2024 [titles translated]. | pp. 177, 180, 182–184 | Official ministry response account, Japanese R14a–c; welfare support, worker matching and historical access/cost measures. |

7 **Evidence-to-design map**

Table S6 links the present specification to conceptual foundations and case evidence. It does not imply that the six domains were empirically derived or that the bundle rules have been validated.

**Table S6. Evidence informing the model’s distinctions and decision rules.**

| **Design issue** | **Evidence or conceptual basis** | **Implication for the specification** |
| --- | --- | --- |
| Six-domain organization | Resource, systems and decision perspectives (15–25); capacity, information and care issues in references 1–14. | Four capacity domains and two feasibility conditions form a proposed synthesis, not an empirically exhaustive taxonomy. |
| Material and fixed damage | Actual local saturation, alternative receiving space (R08b–d) and supply of water, medicines, equipment and oxygen (R10d). | Fixed origin components are distinguished from modifiable (potentially actionable) receiving or supply capacity. |
| Information and capacity | Missing information content, hardware limitations and confirmed receiving access (R02a–b; R08b–c; R12b). | Actual resources, knowledge of resources and communication hardware have distinct roles. |
| Time and chronology | Scheduled deployment limits (R07a), route-related delay (R11a) and observed completion (R08f). | Appraise the relevant horizon or modifiable delay without treating every timestamp as a restriction. |
| Safety/Quality and the frame | Safe placement and clinical grouping (R08a, R08e); facility-function and evacuation evidence (R09a). | Requirements remain in the frame; current capability is appraised separately. A separate S/Q contribution is tested for redundancy. |
| Financial conditions | Hesitation to request support because of uncertain expense eligibility (R13b), accommodation-cost arrangements (R13d), and distinct fee-relief measures (R14c). | Financial exposure or buffering is specific to the decision, policy scope and date. |
| Evidence and decision boundaries | Mission-specific communication (R06b), local resource availability (R12a) and incomplete regional accounts. | Unknown states and different record resolutions are retained; overlapping stages are not independent replications. |
| Minimality and action | Receiving and transport sequence (R08c–f); narrower or incomplete decisions in other records. | Compare smaller explanations and report unresolved or single-restriction results while retaining supported actions. |

8 **Evidence register**

Table S7 contains paraphrases, not verbatim quotations, of the 37 source passages used in the analysis. Reference numbers correspond to the source index in Table S5 and the manuscript reference list. Page numbers follow the cited source’s pagination; separately paginated versions are identified where needed. The “Use and limits” column states how each passage is used; it does not attribute HMFITS-IDM judgments to the source authors.

**Table S7. Source locations, reported content and limits of inference.**

| **Evidence ID / source location** | **Source-reported content (paraphrase)** | **Use and limits** |
| --- | --- | --- |
| R01a \| Ref. 1 pp. 439, 442; Abstract and Results | Retrospective records cover 6,107 patients in 48 affected and 47 backup hospitals. Transport peaked in the first four days; family cars and ambulances were used, with limited helicopter use. | Aggregate movement, not a complete choice record or proof that a particular alternative vehicle was available. |
| R01b \| Ref. 1 p. 443; Discussion, opening and middle paragraphs | Damage, shortages of personnel and supplies, and uncontrolled patient flow constrained affected hospitals. The authors describe evacuation of existing inpatients to accommodate new admissions. | Distinct physical, workforce and coordination restrictions; no basis for excluding Material across the board. |
| R02a \| Ref. 2 pp. 255–256; Effects on Medical Care and Table 4 | Hospitals unable to maintain inpatient care needed information on receiving facilities and transport, but communication was insufficient. The reported information needs changed with the response phase. | Informs destination choice; information on local options and exact deadlines is incomplete. |
| R02b \| Ref. 2 pp. 254, 257; Hardware Damage and Discussion | Power loss disabled computers; some hospitals used generators. Telephone overload and damaged infrastructure impaired communication, while other channels were reported to be useful. | Physical channels and information content interact but are not interchangeable; local alternatives require evidence. |
| R03a \| Ref. 3 p. 53; The Great Hanshin-Awaji Earthquake | The review describes hospital-function loss, uneven patient loads relative to available doctors, insufficient information sharing, and late arrival of medical response teams. | Retrospective regional context for HA2, not an independent actor-level record or evidence of a 1995 DMAT option. |
| R03b \| Ref. 3 pp. 55, 60; EMIS and Discussion | EMIS is described as supporting the distribution of teams, supplies and transportation. The review attributes the late transport peak at Camp Kasuminome to a lack of transportation for mobile DMATs and patients in the early phase. It also reports insufficient logistics and accommodation for some teams. | System-level explanatory context; source-attributed delays are not estimated HMFITS interactions. |
| R04a \| Ref. 4 pp. 556–558, 560–562; Introduction, Roles of Headquarters, DMAT Activities | The report describes the later establishment of trained DMATs, information sharing, coordination, transport support, logistics and expense arrangements. | Later system context; expense arrangements do not establish episode-specific Financial buffering. |
| R05a \| Ref. 5 p. 7; First Response by the Ministry of Health, Labour and Welfare | Timely understanding of medical needs was difficult. Hospital care was affected by prolonged power and water outages and fuel shortages; chronic-care and special-care transfers posed coordination challenges. | Broad response context, not a complete record of any one hospital’s decision. |
| R05b \| Ref. 5 p. 8; Response to the Nuclear Power Plant Accident | The ministry reports support for evacuating hospitals and nursing homes, as well as radiation screening, alongside separate food- and water-safety activities. | Public-health and continuity context; food/water regulation is separate from the TMDUH mission. |
| R06a \| Ref. 6 Article-version p. 3; Field experience of DMAT—Missions 2 and 3 | At Sendai, teams could not obtain information on casualty numbers and needs before arrival. In Iwate, the mission shifted toward emergency and primary care, with different equipment and transport needs. | Mission-specific tasks and constraints; does not establish a common bundle across missions. |
| R06b \| Ref. 6 Article-version p. 3; Field experience of DMAT—Mission 4 | The TMDUH team and other DMATs coordinated radiation screening and evacuation of elderly and disabled patients. Protective equipment and dose-meters were used. Almost all communication methods were available and operable at that time. | The availability of operable devices counters any assumption of persistent communication failure, but it does not establish complete information or resource sufficiency. |
| R06c \| Ref. 6 Article-version p. 4; Outcome/Discussion and Table 1 | Because of communication failure, handover took longer, as it had to be done face to face when ambulances arrived. Table 1 of the source distinguishes the communication conditions of the four missions. | Reported link between information and handover time in the early missions; not attributed to Mission 4. |
| R07a \| Ref. 7 pp. 477–478; Introduction and Analysis | Teams normally planned to operate for 48–72 hours, but the response lasted longer. Some teams withdrew when their scheduled activity ended; loss of a team where medical needs remained could cause major confusion. | Basis of GE2’s workforce–duration–continuity problem. Local relief availability and comparative outcomes are unreported. |
| R07b \| Ref. 7 p. 478; Analysis and Results | The report discusses evacuation when utilities or supplies fail, more varied transport requirements, withdrawal conditions, and additional EMIS training. | Training lessons rather than a contemporaneous complete choice record. |
| R08a \| Ref. 8 Advance-publication p. 2; The Kumamoto Earthquake and Subsequent Medical Response | Kumamoto Central Hospital lost water and power and sustained physical damage. The narrative describes about 190 patients outside for more than ten hours; rain was forecast for the evening and securing a safe place was the immediate priority. | KU1 boundary and urgency. Forecast rain is not an exact deadline; 22:00 is a later output. |
| R08b \| Ref. 8 Advance-publication p. 2; paragraphs before Patient Transfers to Other Hospitals | Before noon, local admitting capacity was overwhelmed. Transfer outside the prefecture was needed, but adequate information and communication were lacking. | Separates actual capacity restriction (M) from unknown acceptance (I). |
| R08c \| Ref. 8 Advance-publication pp. 2–3; Patient Transfers to Other Hospitals | The coordination team used a community network to contact Himeno Hospital, about 70 km away and within a two-hour drive. Himeno agreed to accept up to 150 patients; acceptance information had not been adequately established through prefecture-to-prefecture communication. | A documented route to receiving access; journey time does not specify every vehicle’s schedule. |
| R08d \| Ref. 8 Advance-publication p. 3; opening paragraph | An old building with electricity and water was available; the authors also state that the new building and nursing home could have accommodated the transferred patients if the old building had been unavailable. | A source-stated alternative, not an observed counterfactual. Usable space matters, not one uniquely necessary building. |
| R08e \| Ref. 8 Advance-publication p. 3; Patient Transfers to Other Hospitals | Doctors triaged the narrative cohort of 190. Twenty critically ill patients went to other hospitals. Of the remaining patients, 111 were designated for Himeno: 71 by bus and 40 by ambulance, accompanied by five medical staff. Patients were transported by fire-service ambulances, Japan Self-Defense Forces ambulances and private buses. | Actual grouping and differentiated transport, without numerical triage thresholds or tested alternative plans. |
| R08f \| Ref. 8 Advance-publication p. 3; arrival, reception and follow-up paragraphs | The first transport arrived at about 17:00; by 22:00 the transferred patients had been admitted. Receiving staff were mobilized. The report records no adverse transport events, and it describes subsequent daily staff support and the patients’ return after repairs. | Implementation and outcomes remain separate from prior exposure and minimality judgments. |
| R09a \| Ref. 9 pp. 1673–1674, 1687, 1690; Data collection, Evacuations and Table 11 | A questionnaire investigation examined facility damage and functionality. Evacuations were associated with building and critical-system impairment; Table 11 of the source reports facility-level causes and transfers. | Facility-level context; anonymous identifiers are not linked to Kumamoto Central, and engineering scores are not clinical cutoffs. |
| R10a \| Ref. 10 p. 3; 今回の医療活動 | The official report describes EMIS-informed information gathering and more than 1,500 hospital-evacuation transports, with coordination and logistics support. | Regional total, distinct from the 111-person Himeno subgroup; specific EMIS use for KU1 is not established. |
| R10b \| Ref. 10 pp. 7, 11; logistics deployment and coordination | The report identifies a roughly twelve-hour delay after the main shock in requesting a logistics team, as well as initial local confusion where coordinators had not been designated. | Regional coordination limitations, not a matched KU1 staffing inventory. |
| R10c \| Ref. 10 p. 17; handover from DMAT to medical relief teams | The report describes overall continuity of support but also regions where a new headquarters was set up after the DMAT headquarters had completely closed, with confusion in command arrangements while DMATs and medical relief teams coexisted. | Both overall support and local handover difficulties are retained. |
| R10d \| Ref. 10 p. 18; 病院支援（ライフライン等支援） | The prefectural headquarters used EMIS to assess hospital/lifeline status, arranged evacuation when needed, and requested supplies including water, food, medicines, equipment and oxygen. | Documents potentially actionable Material supply or substitution. |
| R10e \| Ref. 10 pp. 19–20; EMIS/BCP and remaining issues | Not all hospitals in Kumamoto Prefecture were registered with EMIS; the headquarters had to screen damage and register hospitals. The safety of evacuation during aftershocks and at night, and the screening of long-term care insurance facilities, remained issues. | Aggregate EMIS activity does not establish complete hospital or welfare coverage. |
| R11a \| Ref. 11 p. 47; Medical Response of Tohoku University Hospital | The team’s 52-km journey from Noto General Hospital to Wajima Municipal Hospital took more than three hours. On the team’s Day 2, snow closed the main road and patient transportation was not completed as planned. On Day 3, the patient transportation team directed other DMATs to transport inpatients using Self-Defense Force aircraft or other means of transport. | Named transport task for NO1; elapsed time alone is not an independently actionable Time restriction. |
| R11b \| Ref. 11 pp. 47–48; Medical Response | Nurses relieved ward staff, tasks were shared, logistics supported the team, and information was handed to successors. Thirty inpatients were transported by a Self-Defense Force aircraft to Fukui Airport on Day 4. | Reported support and transport, without complete aircraft capacities or individual deadlines. |
| R12a \| Ref. 12 p. 5; Establishment of a disaster response headquarters and team formation | Kanazawa Medical University Hospital reports functioning lifelines for the first four days and available beds, space and human resources during the holiday period. | Affirmative buffering at this receiving hospital, not evidence about Wajima or all facilities. |
| R12b \| Ref. 12 p. 5; New approaches in the initial response | The article reports that information was unavailable during patient transport from an affected core hospital and describes establishing a Starlink-based interhospital information-sharing system (KiMMAShi) for use when mobile networks are disconnected. | Information was not uniformly secondary. Deployment timing and use of this system in NO1 are not established. |
| R13a \| Ref. 13 p. 94; II.3(3), 福祉的支援の強化に向けた体制の強化等, 現状と課題 | Even where evacuees’ welfare needs had been identified, shortages of staff and receiving facilities left some people in 1.5-stage shelters. | NO2 capacity-matching problem; resident-level timing and bypass availability remain unknown. |
| R13b \| Ref. 13 p. 94; II.3(3), 現状と課題 | Limited availability of DWATs able to provide an initial response delayed support. Although welfare support in shelters was already eligible for subsidies, uncertainty over whether DWAT activity costs were covered by disaster-relief expenditure sometimes led local governments to hesitate to request support. DWAT’s planned scope did not include people sheltering at home or in vehicles, and welfare-related transport arrangements required additional coordination. | Financial and decision-frame uncertainty at program level, not exposure assigned to every transfer. |
| R13c \| Ref. 13 p. 101; II.3(5), 現状と課題 | Matching evacuees to secondary accommodation according to their needs took time. Initially, sharing aggregated information on transport demand and vehicle availability was sometimes hindered because the prefectural department responsible for receiving that information had not been clearly identified. Some evacuees experienced prolonged stays in 1.5-stage shelters intended for temporary accommodation. | Matching and information-handoff problems at program level, not one universal interaction. |
| R13d \| Ref. 13 p. 101; II.3(5), accommodation subsection | The report states that increasing the accommodation-use cost standard from JPY 7,000 to JPY 10,000 encouraged the lodging industry to secure accommodation. | Source-attributed Finance–capacity relationship, not a causal estimate or current payment rule. |
| R14a \| Ref. 14 p. 177; Section 1 | The ministry reports facility impairment and prolonged shelter-environment burdens; receiving facilities and medical, care and welfare support were arranged for people requiring additional assistance. | Program-level context for NO2. The report mainly describes conditions as of 1 April 2024. |
| R14b \| Ref. 14 pp. 180, 182; DHEAT and DWAT/care-worker dispatch | DHEAT organized and analyzed information to support local coordination. Separately, MHLW and the national social welfare council matched care workers available for dispatch from other prefectures to staffing requests from facilities and support needs at 1.5-stage shelters, to reinforce care provision. | Describes care-worker dispatch matching, not individual resident placement; no matched-resident comparison is available. |
| R14c \| Ref. 14 pp. 183–184; medical insurance response | The ministry describes access without insurance cards and deferred or waived copayments for specified affected people. | Historical buffering arrangements, distinct from request-cost uncertainty in R13b. |

9 **Source discrepancies and scope**

**Table S8. Source differences retained in the analysis.**

| **Source issue** | **Reported difference / scope** | **Treatment** |
| --- | --- | --- |
| Kumamoto patient totals | Reference 8, Table 1 (advance-publication p. 2), gives 200 Kumamoto Central evacuees; the narrative uses about 190. The Himeno subgroup is 111. | Narrative and subgroup counts retain their stated scope; totals are not reconciled. |
| Main-shock time | Reference 8 gives 01:20, whereas reference 9 and reference 10 (p. 4) give 01:25 on 16 April. | The analysis uses the date and the decision boundary around noon. |
| Mission 4 timing | Reference 6 gives nine days after the earthquake in its narrative but Days 11–12 in its Table 1. | Mission identity and conditions are used without assigning a corrected date. |
| Noto year labels | Reference 12 concerns 2024 but includes “2022” in portions of Methods and Discussion. | Those labels are not used to date NO1 or NO2. |
| Population and phase | Reference 10’s regional transport total, reference 8’s subgroup and reference 9’s anonymous facilities differ in scope. Reference 13 is a November review; reference 14 mainly describes conditions as of 1 April. | Counts are not pooled, hospitals are not matched by bed count, and later recommendations are separate from early actions. |

Abbreviations: BCP, business continuity plan; DMAT, Disaster Medical Assistance Team; EMIS, Emergency Medical Information System; DHEAT, Disaster Health Emergency Assistance Team; DWAT, Disaster Welfare Assistance Team; MHLW, Ministry of Health, Labour and Welfare; TMDUH, Tokyo Medical and Dental University Hospital.
